# Tramadol Alleviates Myocardial Injury Induced by Acute Hindlimb Ischemia Reperfusion in Rats

**DOI:** 10.5935/abc.20150059

**Published:** 2015-08

**Authors:** Hamed Ashrafzadeh Takhtfooladi, Adel Haghighi Khiabanian Asl, Mehran Shahzamani, Mohammad Ashrafzadeh Takhtfooladi, Amin Allahverdi, Mohammadreza Khansari

**Affiliations:** 1 Department of Pathobiology, Science and Research Branch, Islamic Azad University, Tehran - Iran; 2 Department of Cardiovascular Surgery, Isfahan University of Medical Sciences, Tehran - Iran; 3 Young Researchers and Elites Club, Science and Research Branch, Islamic Azad University, Tehran - Iran; 4 Department of Surgery, Science and Research Branch, Islamic Azad University, Tehran - Iran; 5 Department of Physiology, Science and Research Branch, Islamic Azad University, Tehran - Iran

**Keywords:** Tramadol/ therapeutic use, Heart Injuries, Heart/physiopathology, Reperfusion Injury, Rats

## Abstract

**Background:**

Organ injury occurs not only during periods of ischemia but also during reperfusion. It is known that ischemia reperfusion (IR) causes both remote organ and local injuries.

**Objective:**

This study evaluated the effects of tramadol on the heart as a remote organ after acute hindlimb IR.

**Methods:**

Thirty healthy mature male Wistar rats were allocated randomly into three groups: Group I (sham), Group II (IR), and Group III (IR + tramadol). Ischemia was induced in anesthetized rats by left femoral artery clamping for 3 h, followed by 3 h of reperfusion. Tramadol (20 mg/kg, intravenous) was administered immediately prior to reperfusion. At the end of the reperfusion, animals were euthanized, and hearts were harvested for histological and biochemical examination.

**Results:**

The levels of superoxide dismutase (SOD), catalase (CAT), and glutathione peroxidase (GPx) were higher in Groups I and III than those in Group II (p < 0.05). In comparison with other groups, tissue malondialdehyde (MDA) levels in Group II were significantly increased (p < 0.05), and this increase was prevented by tramadol. Histopathological changes, including microscopic bleeding, edema, neutrophil infiltration, and necrosis, were scored. The total injuryscore in Group III was significantly decreased (p < 0.05) compared with Group II.

**Conclusion:**

From the histological and biochemical perspectives, treatment with tramadol alleviated the myocardial injuries induced by skeletal muscle IR in this experimental model.

## Introduction

Restoration of blood flow after a period of ischemia causes ischemia reperfusion (IR) injury. IR injury is a serious clinical problem that occurs in many diseases and surgeries, such as limb orthopedic surgery, organ transplantation, cardiopulmonary bypass, and hypovolaemic shock^1,2^. During IR, tissues are subjected to destructive proinflammatory cytokines and reactive oxygen species released by inflammatory cells, leading to inflammatory injury and cell apoptosis^3,4^. IR also affects the secondary organs, including liver^5^, heart^6^, kidney^7^, lung^8^, and even causes multiple organ failure, which is a common cause of mortality. Therefore, antioxidative, anti-inflammatory, and antiapoptotic agents to attenuate multiple organ injury induced by IR are urgently required.

Various investigators have demonstrated that the opioid pathway is involved in tissue preservation during hypoxia or ischemia, and this protection is mediated via the delta opioid receptor^9,10^. It has been shown that morphine has cardioprotective effects during IR^11,12^. Factors, such as respiratory depression and histamine release, are disadvantages of using morphine in the postoperative period of open heart surgery^13^.

Tramadol is a narcotic-like pain reliever drug as it has an unusual mechanism of action involving opioid, noradrenaline, and serotonin (5-hydroxytryptamine) systems of analgesia. It is certainly useful in the treatment of chronic and acute pain. Although it does not cause respiratory depression, the problems of nausea when used in clinically effective analgesic doses for severe pain and the risk of intra-operative awareness may represent significant disadvantages of tramadol^14^. Recent research discloses that tramadol decreases lipid peroxidation and regulates noradrenalin uptake; therefore, these therapeutic properties are used for the management of myocardial ischemia^15^.

In the past few years, the administration of tramadol was shown to protect against IR injuries in local and remote organs^15-18^. However, the role of tramadol in reducing injury in the myocardium after hindlimb IR has not been addressed yet. In this study, the effect of tramadol on myocardial injury after hindlimb IR was assessed by biochemical and histological changes in rats.

## Methods

Thirty healthy mature male Wistar rats weighing 250–300 g were purchased from the Pasteur Institute of Iran. All experimental procedures and protocols used in this investigation were reviewed and approved by the Committee of Ethics in Research with Animals at the Islamic Azad University Faculty of Veterinary Medicine. They were kept under constant room temperature of 20–22°C, relative humidity of 50%–60%, 12 h/12 h light/dark cycle, with *ad libitum* access to filtrated tap water and commercial food and were placed in individual plastic cages with soft bedding.

### Experimental groups

The rats were randomly divided into three experimental groups of ten rats each (of these ten, five were used for biochemical assays and five for histological analysis): Group I (sham group) was subjected to all procedures, except arterial occlusion. The animals received 2 mL of 0.9% saline via the jugular vein. Group II (IR group) was subjected to IR. Two milliliters of 0.9% saline was administered immediately prior to the reperfusion period. Group III (IR + tramadol group) was subjected to IR. A solution of 20 mg/kg tramadol^16^ in 0.9% saline solution was administered, with a total volume of 2 mL.

### Anesthesia

The rats were weighed and anesthetized using an intramuscular injection of ketamine hydrochloride 10% and xylazine hydrochloride 2% (50 mg/kg and 10 mg/kg, respectively).

### Surgery

After induction of anesthesia, the animals were placed on a board, in a dorsal, recumbent position, with their thoracic and pelvic limbs immobilized with adhesive tape. The jugular vein was isolated and catheterized for the administration of heparin, tramadol, and normal saline. The left hindlimb was prepared for sterile surgery. A skin incision was made on medial surface of the left hindlimb and femoral artery was isolated and was clamped with a non-traumatic clamp for 3 h and followed by 3 h of reperfusion. Prior to the occlusion of the femoral artery, 250 IU heparin^17^ was administered via the jugular vein in order to prevent clotting. Rats were maintained in a dorsal, recumbent position and kept anesthetized (additional doses were given in case of necessity) throughout the duration of the ischemic period. Body temperature was maintained with a heating pad and monitored using a rectal thermometer. The vascular forceps was removed and the surgical site was routinely closed with 3/0 polypropylene sutures following the ischemic period. Subjects in Group I underwent a surgical procedure similar to the other groups but the femoral artery was not occluded.

### Specimen collection

At the end of the trial, rats were euthanized with an overdose of pentobarbital injection (300 mg/kg, intraperitoneal) and the hearts were rapidly excised.

### Histological analysis

For histological analysis, the hearts were fixed with 10% formalin and then embedded in paraffin and sectioned into 5-μm thick sections and stained with hematoxylin and eosin (H&E). The sections were examined in a semiquantitative manner, using 250× and 400× magnifications under a light microscope by a pathologist who was blinded to the experiment and data. The histological parameters, such as microscopic bleeding, edema, neutrophil infiltration, and necrosis, were scored according to the classification of Papoutsidakis et al.^19^ as shown in Table 1. Approximately ten fields of view were examined under each magnification. The total histological score for each specimen was determined by the sum of all the partial scores.

**Table 1:** Histological grading (Papoutsidakis et al.)

	**0**	**1**	**2**	**3**	**Magnification**
Necrosis	None or 1-3 dead cells in < 3 FOV	≤ 3 dead cells per FOV in at least 3 FOV or 4-6 cells in no more than 3 FOV	4-6 dead cells per FOV in at least 4 FOV or > 6 cells in no more than 3 FOV	> 6 dead cells in at least 4 FOV	400×
Polymorphonuclear leucocytes	None or 1-3 cells in < 3 FOV	≤ 3 cells per FOV in at least 3 FOV or 4-6 cells in no more than 3 FOV	4-6 cells per FOV in at least 4 FOV or > 6 cells in no more than 3 FOV	> 6 cells in at least 4 FOV	400×
Eosinophils	None or 1-3 cells in < 3 FOV	≤ 3 cells per FOV in at least 3 FOV or 4-6 cells in no more than 3 FOV	4-6 cells per FOV in at least 4 FOV or > 6 cells in no more than 3 FOV	> 6 cells in at least 4 FOV	400×
Loss of striation	None or 1-5 cells in < 3 FOV	≤ 5 cells per FOV in at least 3 FOV or 5-10 cells in no more than 3 FOV	5-10 cells per FOV in at least 4 FOV or > 6 cells in no more than 3 FOV	> 10 cells in at least 4 FOV	400×
Edema	None	< 10% of FOV in at least 3 FOV or > 10% in < 3 FOV	10%-30% of FOV in at least 3 FOV or > 30% in < 3 FOV	> 30% of FOV in at least 3 FOV	250×
Microscopic bleeding	None	Present in <10% of FOV in at least 3 FOV or > 10% in < 3 FOV	Present in 10%-30% of FOV in at least 3 FOV or > 30% in < 3 FOV	Present in > 30% of FOV in at least 3 FOV	250×

FOV: fields of view

### Biochemical assays

Evidence of oxidative stress was determined from heart tissue homogenates using glutathione peroxidase (GPx), catalase (CAT), and superoxide dismutase (SOD) activities and the levels of malondialdehyde (MDA). Each heart was stored separately at −80°C until analysis. The tissues were homogenized in 0.1 M phospate buffer (pH 7.4) with an Ultra Turrax homogenizer. The homogenates were centrifuged at 5000 rpm at 4°C for 10 min; the supernatants were removed and assayed for MDA, GPx, and SOD activities. Tissue GPx and SOD activities were measured with a Hitachi 917 autoanalyser using commercial kits. SOD and GPx activities were expressed as U/mg protein in tissue samples. Tissue MDA levels were determined by the thiobarbituric acid method of Okhawa et al.^20^ MDA levels were expressed as nmol/mg protein in tissue samples. CAT activities were determined by measuring the decrease in hydrogen peroxide concentration at 230 nm by the method of Beutler^21^. CAT activity was expressed U/mg protein in tissue samples.

### Statistical analysis

Data were analyzed using SPSS statistical software package (version 18). Distribution of the groups was analyzed with one sample Kolmogorov–Smirnov test. The results were analyzed using analysis of variance for comparing multiple means (ANOVA) with post-hoc test analysis. Biochemical data were tested using the Kruskal–Wallis nonparametric test. Data are shown as the mean ± standard deviation and the significance level was 5%.

## Results

The experimental procedure was well tolerated and no animals died during the experiment.

### Biochemical results

**Figure 1:**
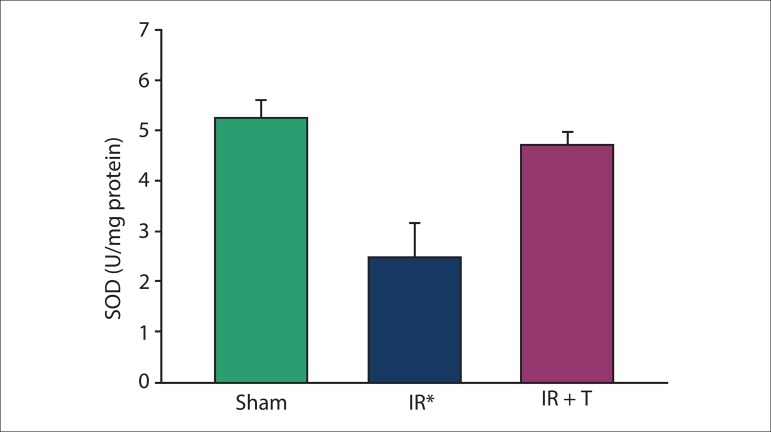
Superoxide dismutase (SOD; U/mg protein) in heart tissue between the groups studied. IR: ischemia reperfusion; and IR + T: ischemia reperfusion + tramadol. Data were expressed as mean ± SD. *: The significant digits in all group were p < 0.001.

**Figure 2:**
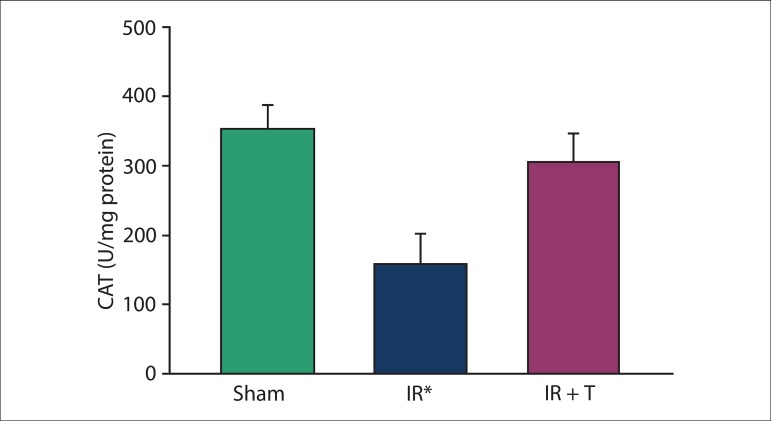
Catalase (CAT; U/mg protein) in heart tissue between the groups studied. IR: ischemia reperfusion and IR + T: ischemia reperfusion + tramadol. Data were expressed as mean ± SD. *: The significant digits in all group were p < 0.001

**Figure 3:**
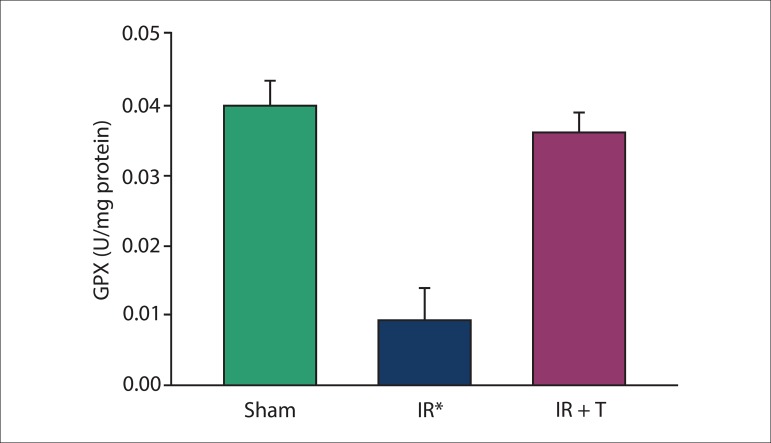
Glutathione peroxidase (GPX; U/mg protein) in heart tissue between the groups studied. IR: ischemia reperfusion and IR + T: ischemia reperfusion + tramadol. Data were expressed as mean ± SD. *: The significant digits in all group were p < 0.001

**Figure 4:**
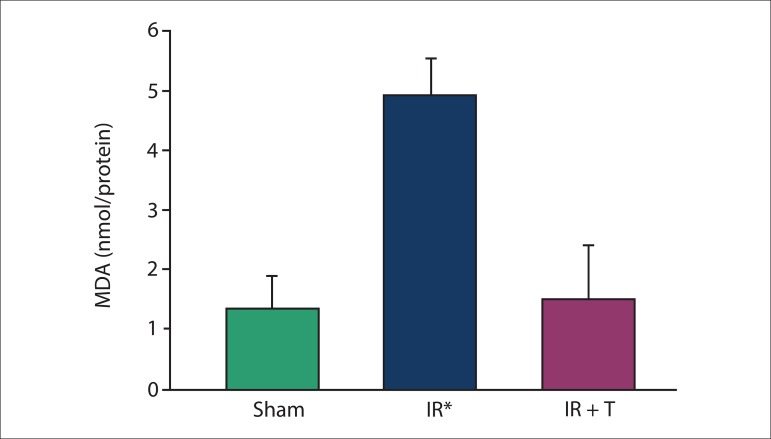
Malendialdehyde (MDA; nmol/mg protein) in heart tissue between the groups studied. IR: ischemia reperfusion and IR + T: ischemia reperfusion + tramadol. Data were expressed as mean ± SD. *: The significant digits in all group were p < 0.001

SOD, CAT, GPx, and MDA levels were measured in the heart tissues after 3 h of reperfusion. The levels of SOD, CAT, and GPx were significantly lower in Group II than those in the other groups (Figures 1–3). The reductions in the levels of these molecules were reversed by intravenous injection of tramadol. In comparison with other groups, tissue MDA levels in group II were significantly increased (Figure 4) and this was prevented by tramadol.

### Histological results

**Figure 5:**
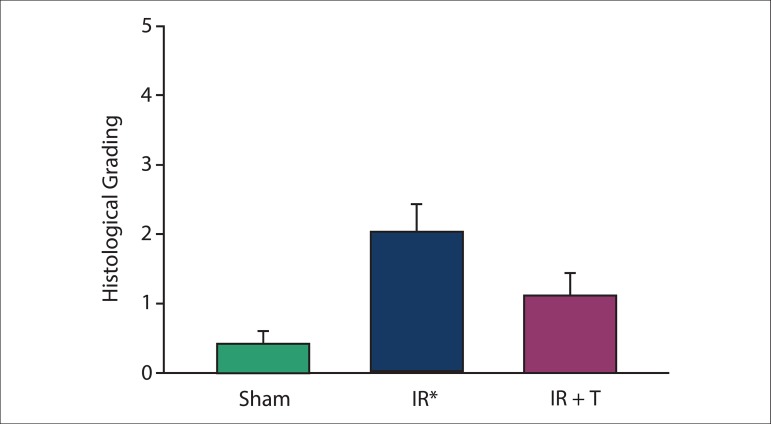
Histological grading between the groups studied. IR: ischemia reperfusion and IR + T: ischemia reperfusion + tramadol. Data were expressed as mean ± SD. *: The significant digits in all group were p < 0.001

**Figure 6:**
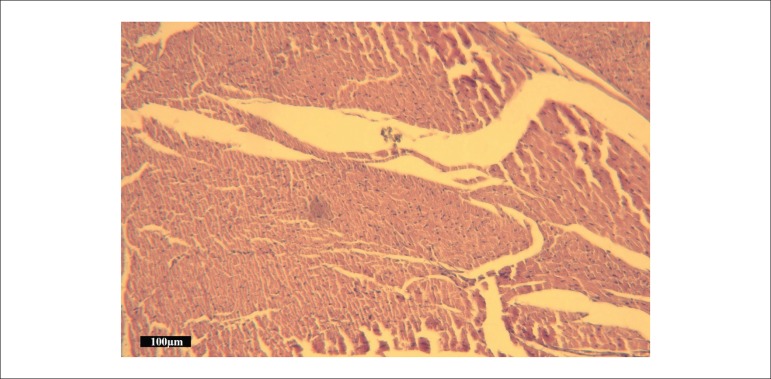
Photomicrograph of myocardium in the ischemia reperfusion group showing coagulative necrosis. Muscle cells with pyknotic nuclei were stained more deeply with eosin in the area of coagulative necrosis (hematoxylin and eosin staining, bar = 100 μm)

**Figure 7:**
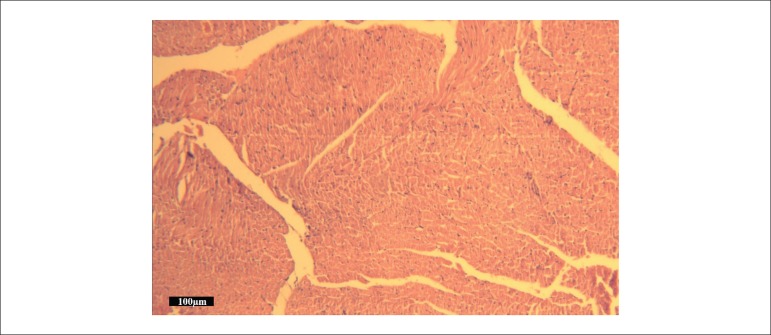
Representative photomicrograph of myocardium in the ischemia reperfusion + tramadol group showing nearly normal structure (hematoxylin and eosin staining, bar = 100 μm)

Histopathological changes, including microscopic bleeding, edema, neutrophil infiltration, and coagulative necrosis, were scored. The total injury score in Group III was significantly decreased compared with Group II (Figure 5). Representative H&E-stained microscopic images of myocardial tissue from Groups II and III are presented in Figures 6 and 7, respectively.

## Discussion

The local and remote consequences of limb IR injury continue to be a serious clinical problem for general vascular surgeons, interventional radiologists, and cardiologists. Reperfusion of the skeletal muscle causes activation and adhesion of polymorphonuclear neutrophils, with the release of proinflammatory substances and the formation of free radicals, which include nitrogen-derived reactive nitrogen species and oxygen-derived reactive oxygen species, such as superoxide, peroxide, and hydroxyl radicals^22-24^. In addition, the proinflammatory and injurious factors activated in large amounts after skeletal muscle IR injury circulate via both the venous and lymph systems and induce distant organ injury^25^. This distant organ injury may be a component of systemic inflammatory response syndrome, acute respiratory distress syndrome, or multi‑organ dysfunction syndrome, which are initially triggered by muscle-derived inflammatory mediators^26^.

As far as we know, there are only a few reports demonstrating remote myocardial injury following skeletal muscle IR injury.^8^ The results of Takhtfooladi et al.^27^ indicated that hindlimb IR induces severe myocardial damage and that N-acetylcysteine has protective effects on the myocardium after hindlimb IR. Their data supported the concept that temporary occlusion of the femoral artery induced myocardial injury in rats^27^.

Previous studies have shown that the use of tramadol after IR in animals attenuated the oxidative injuries. Nagakannan et al.^28^ demonstrated the neuroprotective effect of tramadol against transient forebrain ischemia in rats. Tramadol provides a cardioprotective effect against myocardial IR in isolated rat hearts^15^. Wagner et al.^29^ suggested that tramadol given to humans in high doses actually caused myocardial injury, with increased troponin 1 and decreased inducible nitric oxide synthases expression, possibly due to the systemic undesirable serotonergic effect on diseased coronary arteries.

A recent study showed that ischemia for 2 h was sufficient to obtain a considerable degree of injury in skeletal muscles and the intravenous injection of 20 mg/kg tramadol prevented this deleterious effect^16^. Similarly, tramadol at a similar dose was found to be beneficial on lung injuries induced by skeletal muscle IR when femoral artery clamping was applied^17^. Furthermore, tramadol (20 mg/kg) was determined to be protective against cerebral injuries caused by hindlimb IR in rats^18^. There is growing evidence regarding tramadol’s beneficial effects in ameliorating IR; however, its role in reducing the damage in heart tissue after skeletal muscle IR has not been addressed yet.

In our study, the antioxidant potential of tramadol was investigated using MDA, GPx, CAT, and SOD contents in myocardial tissue following acute hindlimb IR. The MDA level is a marker of tissue lipid peroxidation. The amount of MDA accumulation in tissue is an index of the extent of lipid peroxidation and oxidative stress^15,30^. The lower levels of MDA observed in the group receiving tramadol compared with the IR group supports the hypothesis that tramadol may reduce oxidative stress by scavenging peroxyl radicals. GPx activity is known to depend on reduced levels of glutathione, glutathione transferase, and glutathione reductase. Activities of these enzymes play an essential role in the cellular defense against free radicals^15,30^. Data regarding SOD support a possible antioxidant effect of tramadol. The decreased levels of MDA and elevated levels of SOD activity in tissues may be evidence of decreased lipid peroxidation and increased antioxidant capacity.

The analysis of the myocardium under light microscopy revealed the presence of more edema, neutrophil infiltration, and coagulative necrosis in Group II than in Group III; this shows tramadol’s tendency to attenuate these injuries, a trend that has statistical significance. This observation was supported by Takhtfooladi et al.^27^, who demonstrated that temporary occlusion of the femoral artery in rats resulted in histological changes.

## Conclusion

The results of this study confirmed that the administration of tramadol significantly decreased myocardial injuries induced by hindlimb IR. This protective effect of tramadol is probably ascribed to anti-inflammatory activity. We underscore the necessity of human studies with tramadol that may be beneficial in preventing remote organ injury, particularly during surgical interventions.
